# Influence of the Polymer Binder Composition on the Charge Transfer Resistance, Morphology, and Crystallinity of LiFePO_4_ Electrodes Revealed by Electrochemical Impedance Spectroscopy and Grazing Incidence Small‐ and Wide‐Angle X‐ray Scattering

**DOI:** 10.1002/smsc.202400154

**Published:** 2024-08-10

**Authors:** Fabian A. C. Apfelbeck, Julian E. Heger, Tianle Zheng, Tianfu Guan, Matthias Schwartzkopf, Stephan V. Roth, Peter Müller‐Buschbaum

**Affiliations:** ^1^ TUM School of Natural Sciences Department of Physics Chair for Functional Materials Technical University of Munich James‐Franck‐Str. 1 85748 Garching Germany; ^2^ Deutsches Elektronen‐Synchrotron DESY Notkestr. 85 22607 Hamburg Germany; ^3^ Department of Fibre and Polymer Technology Royal Institute of Technology KTH Teknikringen 34‐35 Stockholm 100 44 Sweden

**Keywords:** electrochemical impedance spectroscopy, GISAXS, GIWAXS, lithium iron phosphate, lithium‐ion battery

## Abstract

Electrode materials for application in lithium‐ion batteries are commonly probed by X‐ray diffraction (XRD) to investigate their crystalline structure. Grazing incidence wide‐angle X‐ray scattering (GIWAXS) is an extension to XRD since in‐plane structures are also accessible. Additionally, with grazing incidence small‐angle X‐ray scattering (GISAXS), morphological information on the nanoscale can be revealed. In this work, the nanostructure of battery electrodes, which consist of lithium iron phosphate (LiFePO_4_) as active material, carbon black (CB) as conducting agent, and the polymeric binders polyvinylidenefluoride (PVDF) and poly((trifluoromethane) sulfonimide lithium styrene) (PSTFSILi) is studied by performing GISAXS and GIWAXS. The chemical nature of the binder is tuned by blending PVDF and PSTFSILi. Specifically, a series of LiFePO_4_ electrodes with polymer blends of the common, non‐conducting PVDF and the single‐ion conducting PSTFSILi with different weight ratios as binders is investigated to understand the influence of the binder on the structure of the electrode in detail. Scanning electron microscopy (SEM) and electrochemical impedance spectroscopy (EIS) complement these studies to correlate the morphology and structure with the electrochemical behavior. It is found that LiFePO_4_ crystallites do not exhibit any preferred orientation with respect to the substrate, irrespective of the binder composition, but their size depends on the binder composition.

## Graphical abstract

The influence of the binder containing polyvinylidenefluoride and poly((trifluoromethane) sulfonimide lithium styrene) on the structure of the battery electrodes, which consist of lithium iron phosphate and carbon black is studied by correlating morphology and electrochemical results.

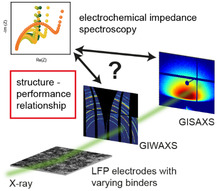

## 1 Introduction

Since its development by Goodenough in 1997,^[^ ^1^ ^]^ lithium iron phosphate has been a widely used active material for electrodes in lithium‐ion batteries, especially in electric vehicles, due to its high safety and thermal stability, flat discharge characteristics, low costs, and environmental friendliness.^[^ ^2^, ^3^ ^]^ In particular, the electrode slurries, consisting of a viscous mixture of the active material, conductive additive, and polymeric binder, are coated onto metallic current collectors by either blade‐coating or slot‐die coating and further processed into the specific battery format.^[^ ^4^ ^]^ The electrode fabrication process is highly crucial as it has an impact on important electrode properties such as the porosity, tortuosity, or effective transport coefficient. However, not only the manufacturing steps and thus controllable engineering parameters (e.g., mixing time, spread velocity, or drying rate^[^ ^5^ ^]^) but also the intrinsic structure on the mesoscale, meaning from nanometer to micrometer, influences the performance of electrodes and, hence, the overall lithium‐ion battery, as elucidated below.

It is well known that the electrochemical performance of LiFePO_4_ batteries depends on its crystalline structure.^[^ ^6^ ^]^ Furthermore, Kim et al. demonstrated, by applying a magnetic field to LiFePO_4_ electrodes, that the orientation of the LiFePO_4_ crystals with respect to the substrate significantly affects the electrochemical behavior.^[^ ^7^ ^]^ Additionally, the LiFePO_4_ particle size affects the ion propagation paths, and a size reduction can effectively improve the charge transport efficiency. Furthermore, the LiFePO_4_ particle morphology can affect the fast‐charging performance.^[^ ^8^ ^]^ The fabrication of the LiFePO_4_ particles with the desired properties is often accompanied by complex synthesis routes utilizing either solid‐state or solution‐based methods.^[^ ^9^, ^10^ ^]^

In addition to the properties of the particles of the active material, the nature of the polymeric binder significantly influences the electrochemical behavior of LiFePO_4_ cathodes.^[^ ^11^ ^]^ In particular, Shi et al. showed that a polymer blend of polyvinylidenefluoride (PVDF) and lithiated poly(perfluoroalkyl sulfonyl)imides (PFSILi, 1:1 wt%) as a polymeric binder reduces interfacial contact resistances and improves the overall performance of LiFePO_4_‐based batteries at high current densities.^[^ ^12^ ^]^ Especially in polymer‐based batteries, the polymer electrolyte is often used as the electrode binder to reduce interfacial contacts.^[^ ^13^, ^14^, ^15^ ^]^ Thus, tuning the nature of the binder of the electrode represents a simple method to obtain superior electrochemical characteristics.

Generally, binder research strongly focuses on intrinsic polymer properties, such as adhesion, electrical and ionic conductivity, chemical stability, and mechanical strength, to enhance the overall electrochemical performance.^[^ ^16^, ^17^ ^]^ However, the underlying binding mechanism between binders and active material particles is also of great importance and has been described, for example, by Chen et al.^[^ ^18^ ^]^ According to them, mechanical interlocking and interfacial binding forces (intermolecular forces and chemical bonds) are responsible for the interaction between the particle and the so‐called bonded polymer layer. Additionally, adjacent particles are connected by the fixed polymer layer, surrounded by the excessive polymer. In the framework of the present study, the bonding system is systematically tuned by changing the composition of the polymer blend binder, and its influence on LiFePO_4_ is investigated with advanced X‐ray scattering techniques, which are described in the following.

Usually, the crystalline structure of LiFePO_4_ electrodes is studied by either powder XRD or XRD with a theta‐2theta geometry and a point detector (0D). Due to the specular condition for each angle, only information perpendicular to the substrate surface can be obtained (structures along the surface normal). In contrast, with grazing incidence wide‐angle X‐ray scattering (GIWAXS), due to the use of a 2D detector, in‐plane structure information is accessible. In addition to that, the small incident angle in the GIWAXS geometry leads to a large footprint (illuminated area) for the whole selected q‐range. Thus, a large sample volume with high statistics of the electrode films can be probed.^[^ ^19^ ^]^ Besides the crystalline structure information probed with GIWAXS, grazing incidence small‐angle X‐ray scattering (GISAXS) enables an investigation of the morphology on the nanoscale. With this technique, statistical information about the particle shape (form factor = FF), as well as the inter‐domain distance of the particles (structure factor), can be obtained. Due to the penetration of the X‐rays into the film and hence the scattering from the bulk, not only features from the surface but also buried structures in the volume of the electrode are accessible. However, so far, in energy‐related materials fields, the techniques GISAXS and GIWAXS are prominently used to study organic and perovskite solar cells to probe the morphology and crystalline structure, which are strongly connected to the device performance.^[^ ^20^, ^21^, ^22^, ^23^, ^24^, ^25^ ^]^ In contrast, in energy storage applications based on batteries, GISAXS and GIWAXS are only rarely used so far, despite the importance of the morphology, and crystalline structure. For instance, the orientation of crystallites in organic materials plays a crucial role in interfacial charge transfer processes.^[^ ^26^ ^]^ In the field of battery research, charge transfer processes are mostly studied by electrochemical impedance spectroscopy (EIS). However, according to Gaberšček, the benefits of this technique are still not fully harvested.^[^ ^27^ ^]^ Varying the electrode's properties, such as the size of active particles or the nature and contents of additives, combined with the development and establishment of complementary measurement techniques that provide structural information, is a promising way to gain new insights into the behavior of the electrode.

In the present study, we use the neutral PVDF and the polystyrene‐based single‐ion conducting polymer poly((trifluoromethane) sulfonimide lithium styrene) (PSTFSILi) (Figure S2, Supporting Information) to systematically investigate the influence of the binder composition on the inner morphology as well as the crystalline structure of blade‐coated LiFePO_4_ electrode films by GISAXS and GIWAXS. For that, we intentionally make use of non‐carbon‐coated LiFePO_4_. We prepare six electrodes with different binder weight ratios of PVDF and PSTFSILi blends (PVDF:PSTFSILi = 100:0, 80:20, 60:40, 40:60, 20:80, 0:100). The focus of this study is to conduct grazing incidence X‐ray scattering on battery cathode thin films and thus provide a characterization technique for battery electrode research, which represents an extension to conventional methods such as XRD and can help to better understand EIS data.

## 2 Results and Discussion

### 2.1 Surface Morphology

**Figure 1:**
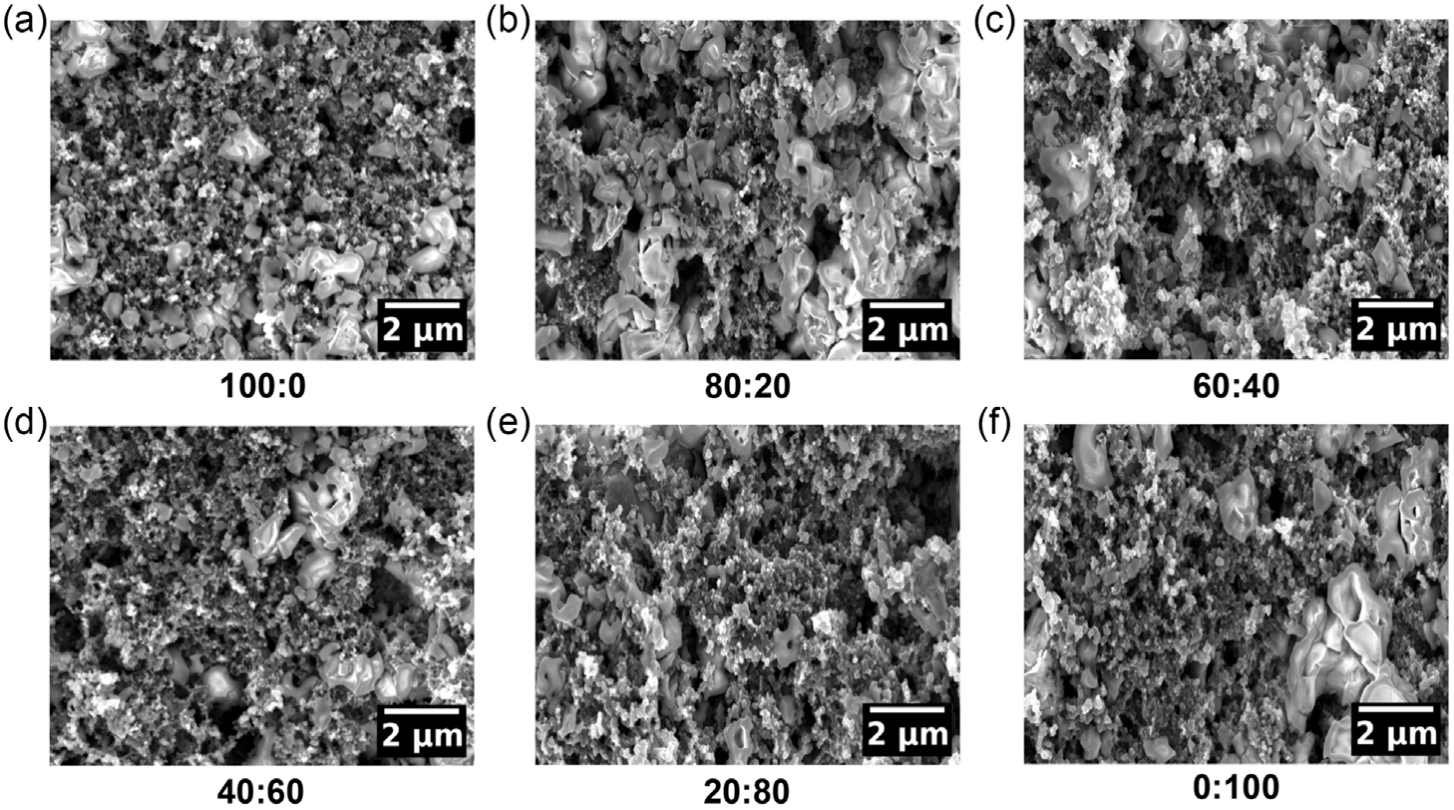
a–f) Surface SEM micrographs of the six LiFePO_4_ electrode films with different binder weight ratios of PVDF and PSTFSILi as indicated.

The surface morphology of the LiFePO_4_ electrodes is investigated by scanning electron microscopy (SEM). **Figure** **1** shows SEM micrographs of the six electrodes with different binder weight ratios. For every electrode, the large particles can be identified as LiFePO_4_, whereas the carbon black particles are the tiny structures that connect the LiFePO_4_ particles. Additionally, for every electrode, voids can be recognized. Overall, the surface of each electrode appears similar, and no significant differences can be recognized. Due to the high surface roughness, inhomogeneous distribution of the material, and the need for statistically relevant analysis of the electrode, surface characterization techniques with a high local resolution, such as atomic force microscopy (AFM) or transmission electron microscopy (TEM) are not performed.

### 2.2 Inner Film Morphology

**Figure 2:**
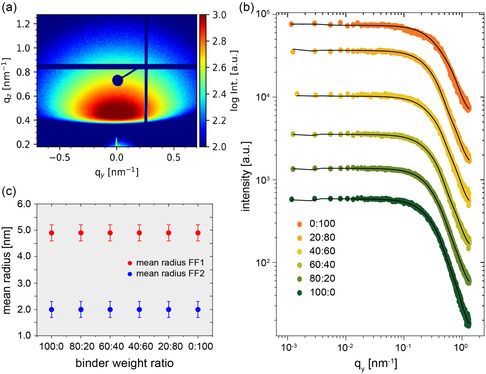
a) Exemplary 2D GISAXS data of a LiFePO_4_ electrode with only PVDF as the binder (100:0). b) Horizontal line cuts (color‐coded) of the 2D GISAXS data with the corresponding fits (black line) of all six electrode films with different binder weight ratios PVDF:PSTFSILi. The cuts are shifted along the *y*‐axis for clarity. c) Mean radius of characteristic in‐plane structures identified from fits to the line cuts as function of the binder weight ratio.

To extend information on the nanoscale morphology of the LiFePO_4_ electrodes from surface to bulk, the samples are investigated by GISAXS. To get this information, firstly, horizontal line cuts are performed on the 2D GISAXS data. Then, these line cuts of the reciprocal space are fitted with a corresponding model, from which real space values such as the radii and distances of the particles are attained. In this model the distribution of the particle sizes follows a Gaussian distribution in the framework of the distorted‐wave Born approximation (DWBA), in which also reflections at the surface are considered in addition to the simple scattering event. A more detailed explanation on the theoretical aspects can be found, for example, in the overview of Hexemer and Müller‐Buschbaum.^[^ ^28^ ^]^ As an example, the 2D GISAXS data of the 100:0 sample is shown in **Figure** **2**a. The absence of a specular peak in the 2D GISAXS data is caused by the high surface roughness of the electrode films. This high roughness is beneficial for applications as electrodes in lithium‐ion batteries since it enhances the surface area and thus favors the penetration of the electrolyte into the electrode. Therefore, contact resistances on the electrode–liquid electrolyte interface are reduced. The scattering contribution of each material can be calculated with the corresponding densities and is given by the scattering length density (SLD). The theoretical value of the SLD for X‐rays at 11.8 keV of LiFePO_4_ is 2.96 × 10^−5^ Å^−2^ (density *ρ* = 3.6 g cm^−3^,^[^ ^29^ ^]^ critical angle *α*_c_ = 0.185°), whereas conductive carbon black and PVDF have more similar SLD values of 1.53 × 10^−5^ Å^−2^ (*ρ* = 1.8 g cm^−3^,^[^ ^30^ ^]^ *α*_c_ = 0.133°) and 1.48 × 10^−5^ Å^−2^ (*ρ* = 1.74 g cm^−3^,^[^ ^31^ ^]^ *α*_c_ = 0.131°), respectively. Accordingly, the position of the Yoneda peak for the corresponding pure materials would have been expected at position of *q*_*z*_(LiFePO_4_) = 0.569 nm^−1^, *q*_*z*_(CB) = 0.515 nm^−1^, and *q*_*z*_(PVDF) = 0.513 nm^−1^. However, in the present study, the Yoneda Peak is broadly smeared out and no well‐defined peaks are seen due to the intermixing of the materials.^[^ ^32^ ^]^ The maximum intensity of the vertical line cut at *q*_*y*_ = 0 nm^−1^ is found at a slightly lower position at *q*_*z*_ = 0.45 nm^−1^ (Figure S3, Supporting Information) independently of the electrode composition.

For the further analysis, horizontal line cuts are taken at this maximum intensity peak position of *q*_*z*_ = 0.45 nm^−1^ for each LiFePO_4_ electrode with the respective weight ratio of PVDF and PSTFSILi (Figure 2b). These cuts do not exhibit any prominent peaks, which indicates a polydisperse structure on the nanoscale without well‐defined nearest‐neighbor distances. The horizontal line cuts are successfully modeled with two cylindrical form factors (FF1 and FF2) with a Gaussian size distribution in the framework of the DWBA. Due to the absence of well‐defined nearest‐neighbor distances, structure factors are not needed in the GISAXS data analysis. In Figure 2b, the black lines display the fits of the horizontal line cuts. For each cylindrical form factor, the extracted value of the radius is plotted in Figure 2c. The small‐sized structures have a radius of (2.0 ± 0.3) nm for each electrode, irrespective of the weight ratio of PVDF and PSTFSILi. This trend also applies to the big‐sized structures with a radius of (4.9 ± 0.3) nm. Considering the surface structure obtained by SEM, in which the polymer can be recognized as an envelope covering the LiFePO_4_ and carbon black particles, it can be assumed that small particles with a diameter of 4 and 9.8 nm agglomerate and form structures with a larger diameter in a polydisperse distribution. Similar observations have been made by Jung et al. who detected aggregated lithium clusters with single particles in the size order of a few nanometers by studying the lithium growth on stainless steel with GISAXS.^[^ ^33^ ^]^ This can be underlined by the findings of Lee et al. who proposed a detailed model of primary and secondary particles for NCM cathode materials, in which primary particles are an aggregation of well‐aligned nanoparticles.^[^ ^34^ ^]^

### 2.3 Crystalline Structure

**Figure 3:**
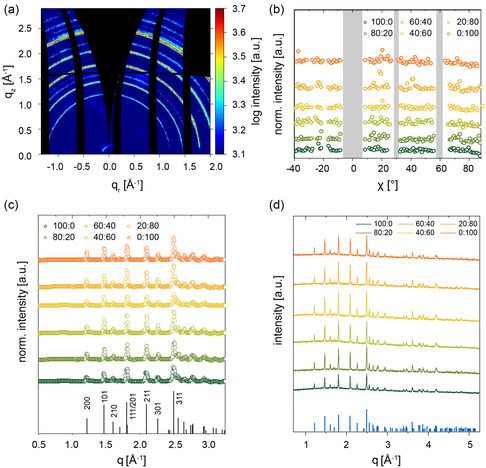
a) Exemplary reshaped 2D GIWAXS data of a LiFePO_4_ electrode with a 100:0 binder composition. Characteristic LiFePO_4_ Bragg reflexes are recognizable. The vertical and horizontal black curved lines represent detector gaps, whereas the area starting from *q*_r_ = 0 Å^−1^, the so‐called “missing wedge”, is inaccessible with GIWAXS. b) Azimuthal tube cuts of the (111)/(201) reflex. The absence of any pronounced intensities along the rings indicates highly disordered crystallites with respect to the substrate. c) Pseudo XRD data extracted from GIWAXS and d) XRD of all six electrode films.

The influence of the blend ratio on the crystalline structure is probed by GIWAXS. As an example, the reshaped 2D GIWAXS data of the LiFePO_4_ electrode with a 100:0 binder composition is shown in **Figure** **3**a. The diffraction reflexes can be indexed to an α‐LiFePO_4_ phase (olivine family cathode material, orthorhombic lattice structure, Pnma space group^[^ ^6^ ^]^) with no indication of a β‐LiFePO_4_‐phase or impurities. Each of the studied LiFePO_4_ electrodes exhibits rings in the 2D GIWAXS data irrespective of the composition, which indicates the presence of small crystallites with a high disorder with respect to the substrate. The intensity along the rings is not homogeneously distributed but shows small intensity fluctuations due to the finite number of crystallites contributing to the scattering signal. To further reveal information about the texture, azimuthal tube cuts of the prominent (111)/(201) reflex at *q* = 1.8 Å^−1^ are performed (Figure 3b). These cuts have a constant intensity over the selected angle range without noticeable peaks for each electrode. This observation confirms the high disorder of the LiFePO_4_ crystallites irrespective of the binder composition.

**Figure 4:**
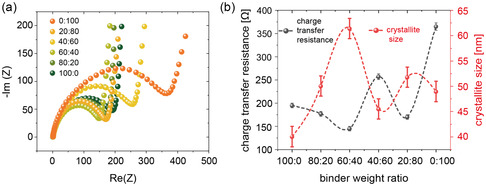
a) Electrochemical impedance spectra of LiFePO_4_ half cells after 24 h of cell fabrication. b) Charge transfer resistances (black dotted line) for all six electrodes extracted from the width of the semicircle and crystallite size (red dotted line) as a function of the binder weight ratio.

Additionally, radial integrations, so‐called pseudo‐XRD (p‐XRD) data, are performed (Figure 3c). The (111)/(201) reflexes are fitted with Voigt functions (Figure S4, Supporting Information). Since the Li‐ion migration pathway is in the [010] direction,^[^ ^35^ ^]^ the (111)/(201) peak, which crystal plane is diagonal in the unit cell, is chosen. A lower boundary of the crystallite size can be estimated from the full‐width at half maximum (FWHM) of the fitted reflex with the Scherrer formalism.^[^ ^20^, ^36^ ^]^ The FWHMs, the corresponding crystallite sizes, and charge transfer resistances are plotted in **Figure** **4**b and S5, Supporting Information. The crystallite size is about (40 ± 2) nm for the LiFePO_4_ electrode with only PVDF and increases by 10 nm to 50 nm with the addition of PSTFSILi (80:20) and reaches a maximum size of (61 ± 2) nm for the 60:40 binder composition. By further increasing the PSTFSILi portion, the crystallite size decreases again to (46 ± 2) nm (40:60). Still, it does not fall below the value of the electrode with only PVDF as the binder, which overall has the lowest value. As we do not observe scattering peaks from PVDF or CB, the (211) reflex at *q* = 2.1 Å^−1^ is also analyzed (Figure S6 and S7, Supporting Information). The FWHM values and, thus, the crystallite size obtained from the (211) peak shows the same trend as the (111)/(201) reflex. In addition to GIWAXS, XRD measurements are performed. The corresponding XRD spectra are shown in Figure 3d. Again, well‐defined Bragg peaks of LiFePO_4_ are seen in good agreement with the GIWAXS findings. However, from the FWHM analysis of the Bragg peaks in the XRD data, no variation in crystallite size is observed (Figure S8 and S9, Supporting Information). Accordingly, along the surface normal, the crystallize sizes are independent of the electrode composition, whereas they differ in the other directions. Such finding underlines the importance of performing GIWAXS studies in addition to the conventional XRD analysis.

### 2.4 Electrochemical Impedance Spectroscopy

EIS measurements are performed on uncycled LiFePO_4_ electrodes to further investigate the influence of the polymer and, hence, variations in crystallite size on the charge transfer resistance. The corresponding Nyquist plots are shown in Figure 4a. Overall, the six electrode films exhibit different widths of the semicircle, which can be attributed to different charge transfer resistances. These differences in charge transfer resistances are not correlated to the orientation of the LiFePO_4_ crystallites. However, for all PVDF‐based electrodes, the evolution of the charge transfer resistance with varying polymer binder composition (Figure 4b) shows a similar trend as the FWHM extracted from the GIWAXS measurements. Thus, the crystallite sizes as seen in GIWAXS need to be considered when explaining the measured charge transfer resistances. In more detail, the charge transfer resistance decreases from *R*_ct_ = (195 ± 5) Ω (100:0) to *R*_ct_ = (177 ± 5) Ω (80:20) and *R*_ct_ = (145 ± 5) Ω (60:40), which might be a combined effect of the increased crystallite size and enhanced ionic conductivity due to the addition of lithium ions with increasing PSTFSILi portion. Interestingly, by further increasing the PSTFSILi content, and hence a predominant portion of the single‐ion conducting polymer in the electrode, the charge transfer resistance of the 40:60 electrode jumps to a rather high value of *R*_ct_ = (257 ± 6) Ω. Surprisingly, the *R*_ct_ drops again for 20:80 to *R*_ct_ = (170 ± 5) Ω, which can be explained by the behavior of the crystallite size. The electrode with only PSTFSILi as the binder exhibits the highest charge transfer resistance. The low ionic conductivity might dominate in this sample, resulting in a high charge transfer resistance despite a crystallite size comparable to the other electrodes.

Interestingly, for the PVDF‐based electrodes, the crystallite size and the charge‐transfer resistance show an opposite trend with varying PSTFSILi content. As the crystallite size gets larger, the charge transfer gets smaller and vice versa (Figure 4b). This finding suggests an improved charge transfer with an increase in the crystallite size. Interestingly, Fan et al. observed a correlation between crystallite size and electrochemical properties, in particular, discharge capacity and rate performance of NMC532 cathodes.^[^ ^37^ ^]^ The adjustability of the crystallite size with the polymer binder is rather unexpected, as the conventional XRD analysis does not indicate such behavior in the first place. Finally, as the surface of the electrode exhibits a high surface roughness (as confirmed with GISAXS), the electrolyte is in contact with the electrode not only from the top but also from all other crystallographic directions. Thus, the charge transfer from the electrolyte into the LiFePO_4_ crystallites can take place from all directions, and hence the use of GIWAXS is required.

Based on these findings, maximizing the crystallite size of the LiFePO_4_ particles combined with a blend of non‐conducting conventional PVDF and an ionic conducting polymeric binder is favorable to decreasing charge transfer resistances and hence optimizing the electrochemical performance. The fact that the binder affects the crystallite size suggests that the polymer and the particles of the active material strongly interact with each other, which has a pronounced effect on the crystalline nature. Whereas weak van der Waals forces dominate between PVDF and LiFePO_4_, electrostatic interactions between the polyelectrolyte PSTFSILi and the particle of the active material prevail. On top of that, the interaction between the two polymer systems and even the carbon black additive emphasizes the complexity of battery electrodes highlighting the necessity of advanced characterization techniques. By simply tuning the composition of the binder with a non‐conducting and single‐ion conducting polymer and thus altering the bonding system, we present a facile and cost‐effective approach to tailor the crystallite size of LiFePO_4_ with commercially available polymers. Future research can make use of the variety of polymers, for example, different backbones or functional groups, etc., to further investigate the interaction between binder and active materials that do not have to be limited to LiFePO_4_.

## 3 Conclusion

In this work, we successfully perform GISAXS and GIWAXS measurements on blade‐coated LiFePO_4_ electrode films with different weight ratios of the polymers PVDF and PSTFSILi as binders. Thus, we demonstrate the feasibility of these techniques on rough battery electrode surfaces. Due to the footprint effect of the grazing incidence technique, a large sample volume is probed for the selected q‐range. A 2D detector collects the scattered signal, which originates from out‐of‐plane and in‐plane structures, in contrast to XRD, where only information along the surface normal is accessible. We show that the crystallites orient randomly irrespective of the binder composition, but their size depends on the polymer binder composition. For PVDF‐based electrodes, the charge transfer resistance obtained from EIS exhibits an opposite trend as the LiFePO_4_ crystallite size. With our work, we show the importance of advanced scattering techniques and aim to establish grazing incidence X‐ray scattering for battery electrode characterization and suggest its use in analyzing novel electrode materials in the future.

## 4 Experimental Section

4.1

4.1.1

##### Sample Preparation

Non‐carbon coated LiFePO_4_ (Sigma Aldrich), carbon black (TMAX Battery Equipment), and the respective polymer blend of PVDF (*M*_W_ = 534 000, Sigma Aldrich) and PSTFSILi (M_w_ = 231 722, Specific Polymers) were mixed in a weight ratio of 8:1:1. First, 62 mg of the composite polymer was completely dissolved in 1.77 g N‐methyl‐2‐pyrrolidon (NMP, Carl Roth GmbH + Co, 99.8%). Subsequently, carbon black (62 mg) and LiFePO_4_ (496 mg) were added and stirred until a homogeneous slurry was obtained. The slurry was blade‐coated on clean ITO glass substrates (Yingkou Shangneng Photoelectric material Co., Ltd.) for SEM and X‐ray experiments. Then, they were dried at T = 80 °C for 12 h. For impedance measurements, the cathode slurry was coated on aluminum foil, and CR2032 coin cells with lithium metal (99.9%, 500 μm, Dongguan Shanshan Battery Materials Co., Ltd) as a reference and counter electrode and 1 M LiPF_6_ in EC/DMC/DEC (Sigma Aldrich) as electrolyte were fabricated in an argon‐filled glovebox (H_2_O < 0.1 ppm, O_2_ < 0.1 ppm).

##### GISAXS/GIWAXS Measurements

Grazing incidence small‐ and wide‐angle X‐ray scattering experiments were performed at the P03 beamline (PETRA III) at DESY, Hamburg.^[^ ^38^ ^]^ A photon energy of 11.8 keV was used. For GISAXS, the SDD was set to 3865 mm using a Pilatus 2 m detector (Dectris Ltd., 172 μm pixel size), and the incidence angle was 0.36°. The software DPDAK was used for horizontal linecuts.^[^ ^39^ ^]^ For GIWAXS, the SDD was set to 297 mm using a LAMBDA 9 m detector (X‐Spectrum GmbH, 55 μm pixel size). The GIWAXS data was analyzed with INSIGHT^[^ ^40^ ^]^ and the reduced data was normalized to the beam intensity. The beam direction was perpendicular to the blade coating direction.

##### SEM

Scanning electron microscope from Zeiss (Gemini NVision 40) with a working distance of around 5 mm and a voltage of 5 kV was chosen.

##### XRD

X‐ray diffraction was performed with a Bruker D8 advance with an energy of 8 keV.

##### EIS

Electrochemical impedance spectroscopy measurements were performed on a BioLogic VMP300 potentiostat at *T* = 25 °C. A frequency range from 1 MHz to 0.1 Hz with an amplitude of 10 mV was chosen.

## Conflict of Interest

The authors declare no conflict of interest.

## Author Contributions

**Fabian A. C. Apfelbeck**: Conceptualization, Formal analysis, Investigation, Methodology, Visualization and Writing – original draft, **Julian E. Heger**: Data curation, Investigation, Methodology, Validation and Writing—review & editing; **Tianle Zheng**, **Tianfu Guan** and **Matthias Schwartzkopf**: Data curation, Investigation, Methodology and Writing—review & editing; **Stephan V. Roth**: Data curation, Investigation, Methodology, Resources and Writing—review & editing; **Peter Müller‐Buschbaum**: Conceptualization, Funding acquisition, Project administration, Resources, Supervision and Writing—review & editing.

## Supporting information

Supplementary Material

## Data Availability

The data that support the findings of this study are available from the corresponding author upon reasonable request.
