# Supplementary material for: Influence of the Polymer Binder Composition on the Charge Transfer Resistance, Morphology, and Crystallinity of LiFePO4 Electrodes Revealed by Electrochemical Impedance Spectroscopy and Grazing Incidence Small‐ and Wide‐Angle X‐ray Scattering
Source: Small Sci. 2024 Aug 10;4(10):2400154. doi: 10.1002/smsc.202400154 (PMC11935098; doi:10.1002/smsc.202400154)
Supplement: Supplementary file 1 — Supplementary Material [file SMSC-4-2400154-s001.pdf]

Supporting Information

Influence of the polymer binder composition on the charge transfer resistance, morphology, and crystallinity of $LiFePO_4$ electrodes revealed by electrochemical impedance spectroscopy and grazing incidence small- and wide-angle X-ray scattering

Fabian A.C. Apfelbeck[1], *Julian E. Heger*[1], *Tianle Zheng*[1], *Tianfu Guan*[1], *Matthias Schwartzkopf*[2], *Stephan V. Roth*[2,3] *and Peter Müller-Buschbaum*[1,*]

[1]TUM School of Natural Sciences, Department of Physics, Chair for Functional Materials, Technical University of Munich, James-Franck-Str. 1, 85748 Garching, Germany

[2]Deutsches Elektronen-Synchrotron DESY, Notkestr. 85, 22607 Hamburg, Germany

[3]Royal Institute of Technology KTH, Teknikringen 34-35, 100 44 Stockholm, Sweden

*Corresponding author. **E-Mail: muellerb@ph.tum.de**

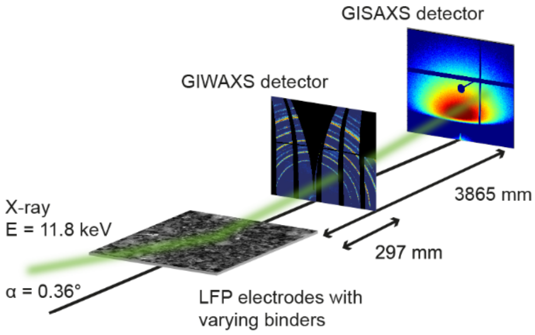

Figure S1. Illustration of the experimental setup.

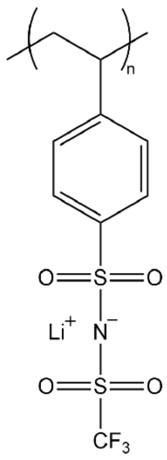

Figure S2. Chemical structure of PSTFSILi.

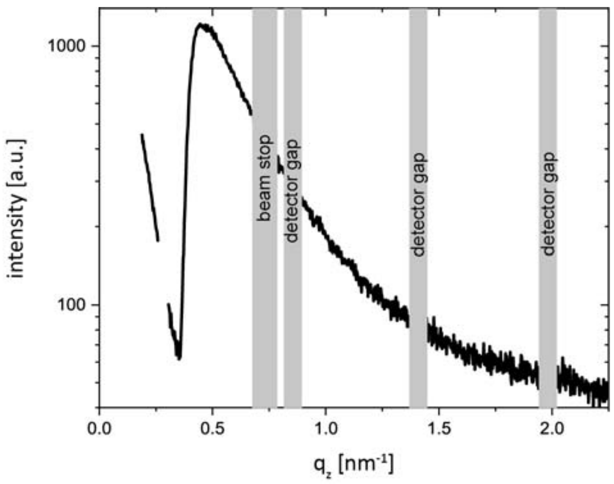

Figure S3.Vertical line cut of the 2D GISAXS data at $q_y = 0$ nm^{-1} for the 100:0 sample. The beam stop position and detector gaps are marked with gray boxes.

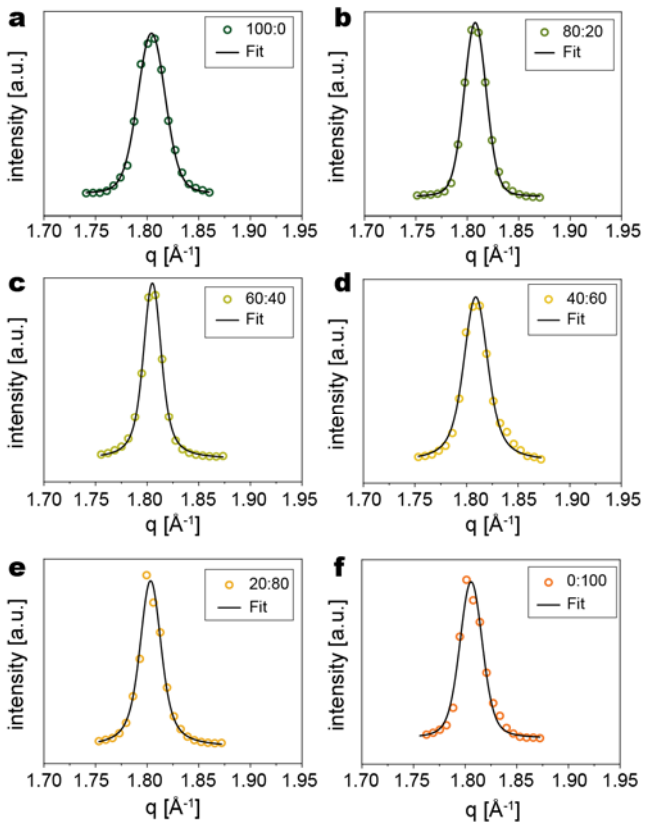

Figure S4. (111)/(201) peaks and fits extracted from the GIWAXS data of the six $LiFePO_4$ electrode films with different binder weight ratios of PVDF and PSTFSILi as indicated.

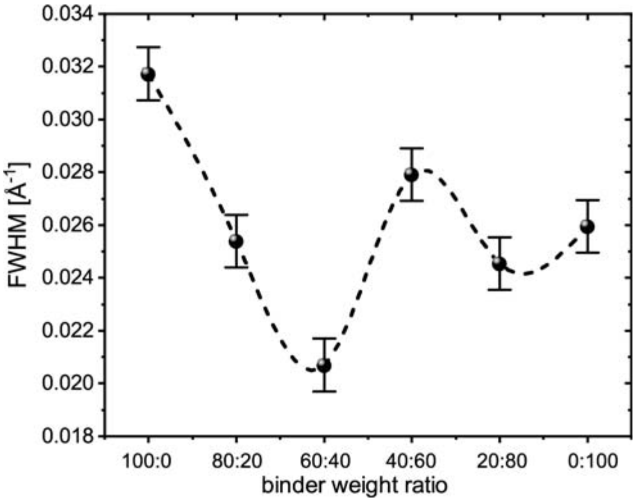

Figure S5. FWHMs from (111)/(201) GIWAXS peak.

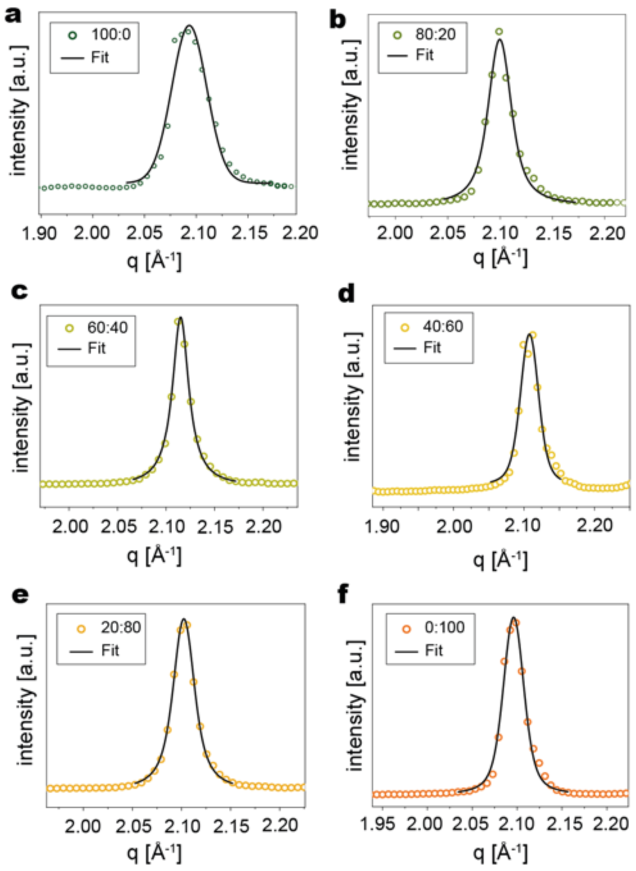

Figure S6. (211) peaks and fits extracted from the GIWAXS data of the six $LiFePO_4$ electrode films with different binder weight ratios of PVDF and PSTFSILi, as indicated.

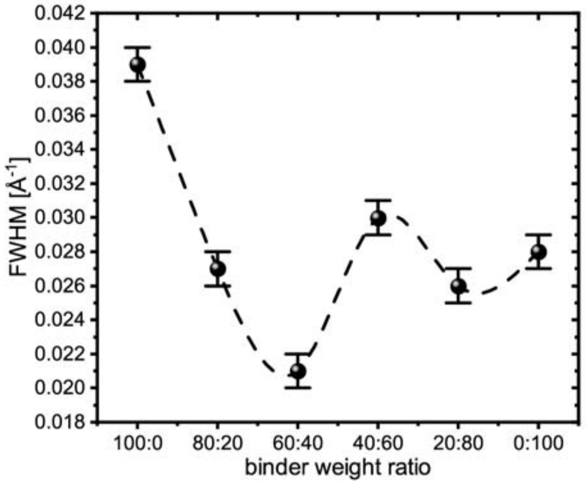

Figure S7. FWHMs from (211) GIWAXS peak.

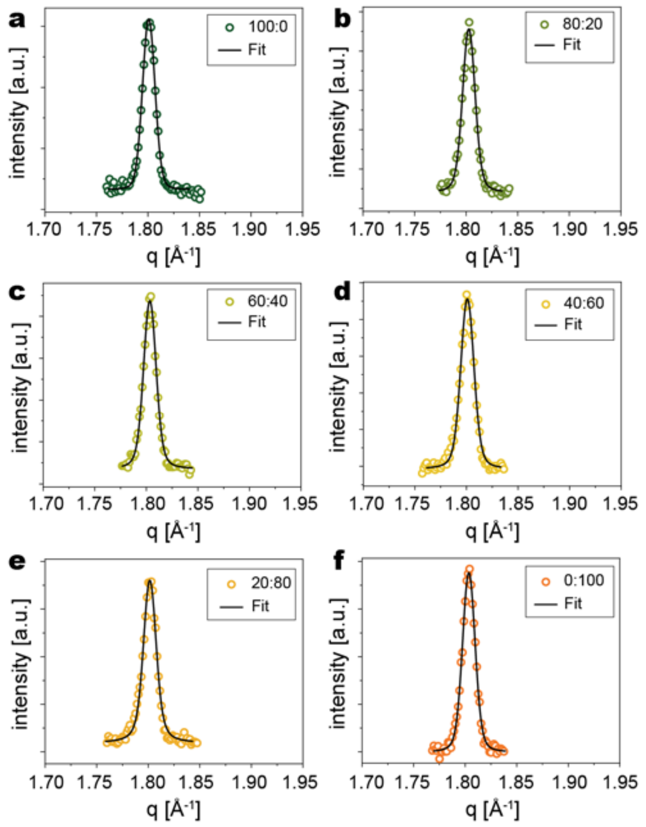

Figure S8. (111)/(201) peaks and corresponding fits (black line) extracted from XRD data of the six $LiFePO_4$ electrode films with different binder weight ratios of PVDF and PSTFSILi, as indicated.

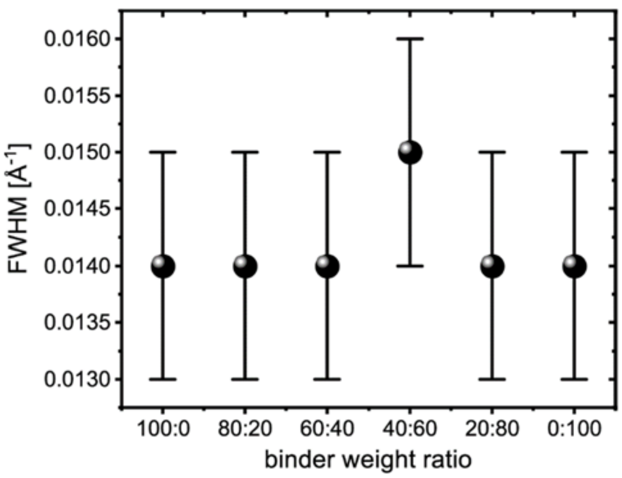

Figure S9. FWHMs from (111)/(201) XRD peak.
